# What Is the Experience of Practitioners in Health, Education or Social Care Roles Following a Death by Suicide? A Qualitative Research Synthesis

**DOI:** 10.3390/ijerph16183293

**Published:** 2019-09-07

**Authors:** Hilary Causer, Kate Muse, Jo Smith, Eleanor Bradley

**Affiliations:** 1 School of Allied Health and Community, University of Worcester, Henwick Grove, Worcester WR2 6AJ, UK; 2 School of Psychology, University of Worcester, Henwick Grove, Worcester WR2 6AJ, UK

**Keywords:** postvention, suicide, suicide loss, suicide bereavement, practitioner, systematic review, qualitative research synthesis

## Abstract

Recent research has highlighted that the number of people impacted by a death by suicide is far greater than previously estimated and includes wider networks beyond close family members. It is important to understand the ways in which suicide impacts different groups within these wider networks so that safe and appropriate postvention support can be developed and delivered. A systematic review in the form of a qualitative research synthesis was undertaken with the aim of addressing the question ‘what are the features of the experiences of workers in health, education or social care roles following the death by suicide of a client, patient, student or service user?’ The analysis developed three categories of themes, ‘Horror, shock and trauma’, ‘Scrutiny, judgement and blame’, and ‘Support, learning and living with’. The mechanisms of absolution and incrimination were perceived to impact upon practitioners’ experiences within social and cultural contexts. Practitioners need to feel prepared for the potential impacts of a suicide and should be offered targeted postvention support to help them in processing their responses and in developing narratives that enable continued safe practice. Postvention responses need to be contextualised socially, culturally and organisationally so that they are sensitive to individual need.

## 1. Introduction

Postvention has been described as activities developed by, with, or for people who are impacted by a death by suicide [1]. The aim of postvention is to facilitate recovery amongst those impacted by a suicide and to prevent adverse outcomes, including suicidal behaviour [1]. In order to appropriately implement postvention, it is vital to accurately identify those who perceive themselves to be impacted by a death by suicide. Authors have debated the definition of ‘impact’ in relation to a death by suicide: Andriessen [1] spoke of a person who has lost a significant other (or loved one) by suicide, and whose life is changed because of the loss; whereas Jordan and McIntosh [2] identify someone who experiences a high level of self-perceived psychological, physical, and/or social distress for a considerable length of time after exposure to a suicide as impacted. The latter definition does not specify the kind of relationship that may exist between the deceased and the person impacted. As such, a broader network of individuals could be considered as having been impacted by a death by suicide. Indeed, it has been estimated that every death by suicide impacts up to 135 people [3]; this includes people drawn from broader networks including health and social care professionals, colleagues, neighbours, social networks in addition to family and close friends. Despite increased recognition of a wider circle of individuals impacted by a death by suicide, postvention research to date has focused narrowly on the experiences of family members and friends after a suicide [4,5]. Although undoubtedly important, it may also be important to consider the wider impact of suicide on those who may potentially be impacted within their working lives and to consider specific postvention needs of this broader group.

Health, social care and education workers form part of a wider network of practitioners who may be impacted by a death by suicide. Studies suggest that 86% of GPs encountered at least one patient suicide in the previous ten years, [6]; 55% of nurses working in psychiatric settings in Japan encountered at least one patient suicide [7]; 43% of psychiatric trainees experienced one or more suicides [8]; nearly 36% of teachers were exposed to at least one student suicide [9]; and 33% of social workers had been exposed to suicide in the course of their job [10]. A recent meta-analysis of population-based studies has shown that exposure to suicide amongst the general population is 4.31% for past-year prevalence and 21.83% lifetime prevalence [11]. This indicates that health, social care and education workers are at an increased risk of exposure to death by suicide at least once during their working lifetime, compared to the general population.

The range of responses that people exposed to suicide may experience following a death by suicide can be considered along a ‘continuum of suicide survivorship’ [12]. This nested model illustrates that people may perceive themselves to be exposed to suicide (i.e., those who knew or, identified with, or came into contact with the individual), affected by suicide (i.e., those who experienced significant distress following exposure), or bereaved by a suicide death (i.e., those who shared a close connection with the deceased and experience a clinically significant negative impact in the short or long term). In principle, health, social care, and education workers exposed to suicide may fall at any point within this continuum. Indeed, Cerel et al. [13] suggest that perceptions of closeness, as described by the suicide survivor, are key to understanding perceived impact of the death, and that those who feel impacted by a death by suicide may include individuals across a range of relationships with the deceased. Furthermore, perceptions of greater closeness and impact relate to higher incidences of depression, anxiety, post traumatic stress disorder (PTSD) and prolonged grief [13]. As such, a number of adverse effects have been reported by health, social care and education practitioners following a death by suicide. These range from professional doubt and fear of legal consequences [14], to feelings of responsibility for the death [15], emotional turmoil and stress reactions [14], and severe distress [16]. For some mental health professionals, post-traumatic responses such as intrusion, avoidance and hyper-arousal have been reported as being so severe and persistent that they fell within a clinical range [17]. Thus, health, social care, and education workers may perceive themselves to be affected or even bereaved by suicide and may also require postvention support.

To develop and implement appropriate postvention support, it is important to better understand the differential experience and impact of suicide on specific sub-groups [18]. Qualitative research is well suited to providing an in-depth account of an individual’s personal experience [19]. Indeed, there is a growing body of qualitative literature that explores health, social care and education worker experiences following a death by suicide [20,21,22,23,24,25,26,27,28,29,30,31]. This literature provides a rich insight into practitioners’ experiences following a death by suicide; however, these findings are dispersed across the literature. This systematic review has been designed to further add to this literature by bringing together knowledge that has been generated by existing independent qualitative studies of health, social care and education practitioners’ experiences following a suicide death to contribute to our understanding of these individuals postvention needs as professional workers.

The research question underpinning this review asks, ‘What are the features of the experience of workers in health, education or social care roles following the death by suicide of a client, patient, student or service user?’

## 2. Materials and Methods

This systematic review took the form of a qualitative research synthesis [32]. Evidence that describes the experiences of health, social care and education professionals following a client death by suicide was reviewed and subjected to an interpretative synthesis [33]. Numbers of qualitative studies have increased over the past few decades, however qualitative research continues to face the challenges presented by a narrative that it lacks validity, value and is difficult to apply to real world settings [34]. Synthesising qualitative studies offers a means of making the most of existing research by examining literature across the breadth of a topic with the aim to generate a greater depth of information [32]. This synthesis brought together a diverse, yet topically connected group of studies with the aim of generating greater depth of knowledge of the experiences of particular groups to a particular event within a particular setting. Knowledge of the particular, in this instance, the individual, their experience and their responses, has an important role in developing best-practice in practice-based disciplines [34]. The enhancing transparency in the reporting of qualitative research statement (ENTREQ) provided a guiding framework in the reporting of this study [35].

### 2.1. Search Strategy

Comprehensive database searches were conducted in February 2018 by H.C. The databases were purposively chosen to screen articles published in psychology, health, nursing and education journals and included PsycInfo, CINAHL, SCOPUS, ERIC and Medline. The reference lists of relevant studies were hand searched for additional references. Database alerts were set up to identify subsequently published studies and an additional study was included in the synthesis in March 2019 [25].

Search words were used as follows:

Medline: Suicide AND [client OR patient OR service user OR student] AND [impact OR effect OR influence OR experience] AND [qualitative research OR qualitative study OR qualitative methods OR interview]

PsycInfo; CINAHL; SCOPUS; ERIC: Suicide AND [client OR patient OR service user OR student] AND [impact OR effect OR influence OR experience]

Searches limiters were used as follows:

Medline:Journal ArticlePsycInfo:Peer Review; Qualitative StudiesCINAHL:Peer ReviewSCOPUS:Journal Article; Peer Review; Qualitative; InterviewERIC:Journal Article; Peer Review

### 2.2. Screening the Literature

**Figure 1:**
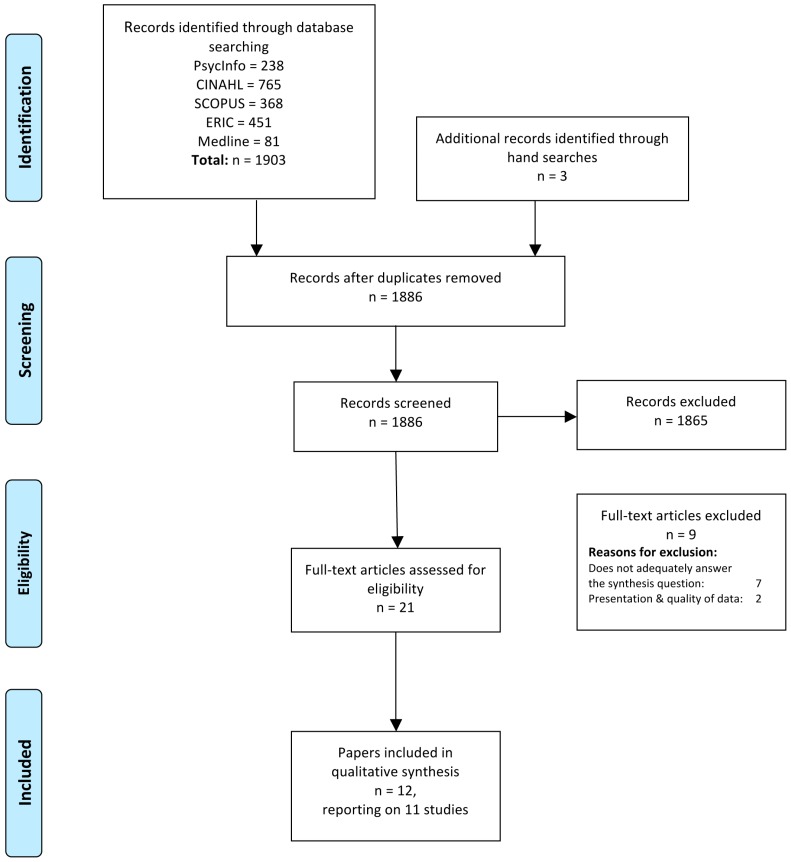
PRISMA flow diagram of the selection process. Adapted from the Preferred Reporting Items for Systematic Review and Meta-analyses (PRISMA) flow diagram [36].

Lead author, H.C., undertook article screening; all the articles were collated and duplicates removed. Remaining articles were screened by (i) title (ii) abstract and (iii) methods, research question and presentation of data and (iv) whether the study question and data usefully contributed toward answering the synthesis question. The screening process is summarised in Figure 1.

### 2.3. Study Eligibility

Studies were included if the article was (i) published in a peer review journal; (ii) published in the English language; (iii) reported on the subjective experience of health, social care or education professionals following a client death by suicide; (iv) data collection and analysis methods were reported as qualitative, or as mixed methods if the qualitative data were reported in a distinct section of the article; Noblit and Hare’s [33] definition of qualitative research as being that which seeks to generate understanding of participants’ subjective experiences and uses an interpretative framework was applied; (v) reported findings were evidenced with the inclusion of verbatim quotes.

Studies were excluded if they (i) employed solely quantitative data collection and analytic methods; (ii) sought participants’ views on the topic of suicide, organizational responses to suicide, participants’ experiences of suicidal ideation or behaviour that did not result in a death by suicide or (iii) were non-empirical case reports or opinion pieces.

### 2.4. Quality Assessment

The study used the Critical Appraisal Skills Programme (CASP) Qualitative Research Checklist [37]. The CASP tool has been subject to evaluation and has been found to take a generic inclusive approach that is helpful when working with a body of literature that includes diverse methods, populations and sample sizes [38].

All of the studies included in the review met all of the criteria in the checklist with the following exceptions: Tillman [29] lacked clarity in describing the process of recruiting participants. Two further studies, Sanders et al., [28] and Ting et al., [30] used participants selected from a larger sample who, in both cases, had participated in a larger quantitative study. Consideration of the relationship between the researcher and the participants was only explicit in Kim [25] who acknowledged that the researcher shared the same profession as the study participants. Five of the studies made no mention of ethical considerations or approval for the research [23,24,28,29,30]. Bohan and Doyle [20] did not report any process of oversight of the analytic process. Taking these exceptions into consideration, there remained demonstrable consistency in quality across all of the studies in relation to their aims, methods and reporting. CASP quality ratings are reported in Supplementary Table S1.

### 2.5. Data Extraction

The studies that addressed the synthesis question and met the inclusion criteria were eclectic in terms of research question and aims, location of study, participant groups, methodology and quality of reporting. Lead author (H.C.) extracted data to inform an overview of the studies consisting of study location; authors and date of publication; qualitative method; participants; study aims or question; methods for qualitative analysis; key themes or domains that were found. The overview is reported in Table 1.

H.C. extracted all data under the headings of Results or Findings and Discussion for the purpose of the synthesis [32]. The inclusion of the authors’ interpretations produces the triple hermeneutic that typifies an interpretative synthesis [32]. Inclusion of the discussion sections as data gave an opportunity to embrace not just the findings of diverse research studies, but also the important work undertaken by diverse researchers in understanding, contextualising and interpreting their findings.

### 2.6. Analysis

Analysis of the data started with an initial comparison of themes across the studies followed by an in-depth coding of the extracted data to generate the synthesis and interpretation [32].

#### 2.6.1. Comparison of Themes

To develop an understanding of how the findings of the studies sat alongside one another, it was helpful to look in greater depth at the themes identified within each of the studies [32]. An iterative reading of the articles was undertaken that included note-taking and reflection in order to identify themes, shared features, but also where they differed. This was the first stage of moving from the idea of working with twelve individual articles toward a sense of working with a large and detailed collective data set. Themes were identified from each article and entered by name on a table. This process enabled the themes to be organised across the articles by commonality and difference. Taking an overview of the themes from each article enabled identification of groups of themes across articles.

Groups of themes that were developed in this process were:

- What happened—experiencing the event
- Emotional response—experiencing the feelings
- Impact on practice—how do I practice now?
- Internal responses/self scrutiny—Am I ok?
- Responses of others—do they blame me?
- Experiences of support—who will look after me?
- Self care/maintenance—can I look after myself?
- Training needs—what do/did I need to know?

#### 2.6.2. Coding, Synthesis and Interpretation

Articles were read in depth and notes were made pertaining to the researcher’s initial impressions regarding the organisation of the findings; inclusion of verbatim data; style of reporting findings and discussion. Initial coding took a descriptive form; codes were noted onto a template and then assimilated into each other to identify concepts and themes that described commonality or differences across articles. This was an iterative process as the researcher moved between data, notes, the groups of themes identified by the article authors and the developing table of codes.

The synthesis and interpretation were developed iteratively by working between the concepts, themes, transcripts and the review question. The researcher sought to move beyond the comparison and aggregation of the results from the included studies toward the revelation and development of an interpretation [32]. Throughout the analytic process, each stage was discussed by the lead and co-authors, providing the opportunity for reflection, testing and checking of categories, themes and concepts as they were developed. It was imperative throughout to stay close to the voices of the participants from across all the published studies.

## 3. Results

The analysis resulted in the development of three categories of themes and concepts. All three categories were evident in the data across all studies:

- Horror, Shock and Trauma
- Scrutiny, Judgement and Blame
- Support, Learning and Living With

Each category contained a number of themes that, in two of the category groups, contained a range of concepts. The relationships between the concepts, themes and categories are illustrated in Table 2.

### 3.1. Horror, Shock and Trauma

The category ‘Horror, shock and trauma’ described the practitioners’ experiences at the moment of and following their client’s death by suicide. It described their immediate cognitive and emotional responses and the psychological and physiological responses that were experienced in the moment and over time. The themes within this category included ‘Witnessing suicide’, ‘Personal responses to suicide’ and ‘Dealing with suicide’. They described three ways in which the practitioner experienced the horror of a client suicide through shock responses and experiences of trauma in the immediate moments following the death (or receiving news of the death) and over the subsequent hours, days and months.

#### 3.1.1. Witnessing Suicide

This theme described the experiences of participants who were present at the moment of, or immediately after a suicide death occurred. These experiences can include multiple aspects of horror; perceptions of the event as horrific; the horror of seeing the suicide happen or of seeing the physical trauma that a suicide has caused the body; the horror of being present with a dying or dead body:

*“I had to do CPR with the inpatient vomiting blood. Bleeding profusely from the inpatient’s head, terror of the death scene made me all of a quiver”*

[31].

‘The horror’ also included experiences of recalling or imagining the event of the suicide:

*“I have incredible nightmares. To this day, at night-time, it’s like his face exploded and all I remember was blood and remnants”*

[22].

There was also the horror that someone known to the practitioner had made the decision to die by suicide:

*“A mother of two small girls, she jumped in front of a train, she wasn’t very old, it was awful.”*

[24].

‘In the moment responses’ to the suicide were felt deeply and included feelings of disbelief, bewilderment and numbness:

*“[I] had trouble conceiving that he had actually followed through”*

[28].

For some practitioners, the fact of suicide presented a challenge to their core professional value to keep people alive, even when faced with the fact of death:

*“to work as a team with all the other nurses to try and save this person”*

[20].

Feelings of ‘shock and trauma’ and of being ‘shaken’ were complex; the sense of being overwhelmed was palpable in the words of some practitioners:

*“I was absolutely stunned and completely and immediately traumatised. I was absolutely shocked.”*

[29].

#### 3.1.2. Responses to Suicide

This theme evidenced the range of emotional responses that practitioners described following a death by suicide. This group of themes reflected the impact of the event on the self of the practitioner and their feelings in response to that impact.

‘Loss and grief’ described practitioner recognition of such feelings and their perceptions of the ways in which the feelings impacted upon them:

*“I could not control my crying. I mean, I was grief-stricken. When I say I came undone that’s when I really let myself open up and sob and cry.”*

[30].

‘Mind and body responses’ also took the form of physiological and intrusive thoughts, sometimes presenting as trauma symptoms and potentially leading to burnout:

*“the night he died, I got deathly ill: I had to go to the emergency room. I thought I was having a heart attack.”*

[30].

#### 3.1.3. Dealing with Suicide

This theme evidenced how the event shaped the ongoing thoughts, experiences and behaviours of practitioners.

Perceptions of ‘connection and closeness’ with the deceased impacted the kinds of feelings practitioners experienced following a death by suicide.

The concepts of ‘absence and distance’ described challenges in building a relationship with a client or the perception of a professional distance that shaped the responses of practitioners:

*“I had a patient who took his life. Something was wrong, but I couldn’t find out what. I couldn’t get him to talk about it … I asked him 17 times if he was depressed, but he said that he was fine. It was terrible.”*

[24].

### 3.2. Scrutiny Judgement and Blame

The category ‘Scrutiny, judgment and blame’ was the largest in terms of number of concepts within it and how many studies evidenced those concepts. It describes the processes of reflective examination of both the event and the practitioners’ role and responsibilities in the context of the event. These processes took place for both the practitioner and for others, including the deceased’s family; colleagues; agencies and organisations; and social and cultural contexts. Themes within this category included ‘Thinking about responsibility’, ‘Having to carry on’, and ‘Dealing with others’. These themes illustrated that practitioners’ experiences spanned the cognitive, emotional and behavioural. Perceptions of incrimination and absolution were evident in the data throughout this category.

#### 3.2.1. Thinking about Responsibility

This theme described the range of cognitive processes experienced by practitioners following a suicide death. Thoughts included those about self as a practitioner; about the person who has died; and about perceptions of other peoples’ thoughts, behaviours and responses.

The question ‘am I responsible?’ was present across all of the studies in the synthesis. The concept of responsibility was integral with practitioners’ job-role; keeping people alive or mentally well is a core value as a practitioner and as such triggered practitioners’ deepest fears:

*“It feels like an incredible responsibility. I know when I started with this woman, she was suicidal on and off; I spent a lot of emotional energy worrying if I screwed up on my decisions.”*

[30].

Some practitioners sought out justification in attempts to absolve themselves of responsibility:

*“I told myself I wasn’t responsible for this – that his social worker was away. I was merely covering her caseload. All in an attempt to distance myself”*

[28].

Practitioners anticipated that others might perceive that they held responsibility:

*“I was just so stunned, and worried what people thought … would think ‘she did a terrible job or else her client wouldn’t have killed herself’”*

[30].

The question of responsibility often took the form of intense self-scrutiny, doubt and self-incrimination by practitioners:

*“I scrutinized like mad if he had – was there something special about him, was he miserable, was there something I completely overlooked – I really tried to rewind the film as well as possible. I really tried to scrutinize if there was anything in that consultation which I ought to have picked up, have caught …”*

[24].

Practitioners often asked themselves ‘what did I miss?’, and in the asking of the question found themselves wrestling with the implications of self-incrimination:

*“… did I give him the medication he then killed himself with?”*

[27].

Fears and feelings arose for practitioners from their concerns about ‘professional failure’ and any subsequent guilt, reprisal and impact on reputation:

*“Well this is an awful failure, I think, as a doctor to have experienced that. It is horrible. I think, there we have actually failed”*

[24].

It was evident that the practitioner felt and feared incrimination by self and others:

*“… a fear that I will be accused of screwing up somehow … I was afraid the [students] wouldn’t ever want to talk to me again. That they would be really angry with me, and that nobody would trust me”*

[22].

Ideas of ‘the autonomous client’ allowed practitioners to recognise the limits of their control over client choices:

*“It’s a fact of life I’m afraid, you can’t stop some people from taking their own lives”*

[27].

In some cases, this idea of autonomy led to frustration for the practitioner:

*“I was pissed [at the client]; felt like why the hell couldn’t they have called me? Why couldn’t they have talked to me?... They have not thought to turn to me?”*

[30].

In other instances, practitioners found a route toward absolution:

*“it’s not my responsibility because if they’re going to kill themselves they’re going to kill themselves. I have absolutely no control over that”*

[30].

Some practitioners sought to understand the autonomous clients’ motivation to die:

*“sometimes a person feels the only way they can attain complete autonomy is through their own demise”*

[28].

In some cases, this led to a sense of understanding and compassion about their clients’ choices:

*“She did what she felt she had to do. She was tired of trying to cope with life and never quite succeeding. Suicide was her first real success in life and she proved to everyone she was capable of doing it”*

[28].

#### 3.2.2. Having to Carry on

This theme described the ways in which a client death by suicide may impact ongoing practice.

Feelings and experiences of ‘aloneness’ and isolation with their experience were evident:

*“I don’t think anybody would be different in the amount of aloneness you feel about it all … it would have been nice to have someone to guide me through all of that”*

[21].

For other practitioners, isolation came about due to the impact of trauma:

*“Clinicians reporting dissociative phenomena were also those who experienced more isolation from colleague support, feeling alone with the trauma of their patient’s suicide and cut off not only from others, but also, in the dissociative experience, from themselves.”*

[29].

‘Issues of control’ were evident. The experience of a client death by suicide left practitioners feeling powerless:

*“there is a lot that is not under our control given the population of clients that is worked with in mental health treatment facilities”*

[28].

For some, control meant containing their emotional response:

*“I’ve been a GP for 30 years and you just have to deal with it and accept it”*

[27].

For other practitioners, work place processes were helpful in supporting them to re-gain a sense of control over the events that occurred:

*“It was very important for all of us involved in his treatment to share the last contacts we had with him and what he had said, what we had said, and where we were left. Not so much pointing the finger but searching for anything that could help us make sense of it”*

[29].

However, for some practitioners the process of understanding the limits of their control was challenging:

*“I think that eventually I had to understand that there is no control in a lot of those situations and in that whole process that I had to take ownership of myself and let everything else go”*

[21].

‘Avoidance strategies’ took different forms but a common theme appeared to be that practitioners were seeking ways of absolving themselves from further risk or future experiences with suicidal clients:

*“We came up with this policy, if a person has been actively suicidal within six months, we send them elsewhere. We used that six months I think to distance ourselves somewhat, to make us less involved in the most at-risk clients. It has impacted my practice. I try to create a little more distance from being that first responder”*

[30].

Avoidance of triggering memories was also evident:

*“I won’t enter the ward the patient lived if it was not really needed, lest recalling the suicide scene and making me nervous”*

[31].

‘Hyper-vigilance’ evidenced practitioners’ perception of having or needing to make increasingly frequent or thorough checks; either with clients who had known risk or those who had no record of risk; practitioner responses could impact upon subsequent practice:

*“when I have a patient who is getting closer to thinking about suicide, my anxiety goes through the roof more than it did before, and I am clear that suicide is just not an option. I’m not open to bargaining … and I demand the patient seek hospitalisation”*

[29].

#### 3.2.3. Dealing with Others

This theme described the ways in which the ideas and behaviours of others are perceived and experienced by practitioners. ‘Others’ may be colleagues, managers, organisations, family of the deceased, and social and cultural contexts. It was evident that some of the most explicit perceptions of incrimination were experienced in the relationships and interactions that practitioners had with third parties.

Experiences within ‘organisations and with colleagues’ evidenced that cultural or behavioural boundaries impacted upon the practitioner’s experiencing of the event from the moment of a client death through to processes of healing:

*“All teachers weren’t caught talking about it for it was a top secret for the school. And the principal forbade the news about it from leaking out to anyone… Suicide brings about school … shame … [t]hat’s why schools tried to hide the news”*

[25].

It was evident that the nature of some job-roles called upon practitioners to provide support for others who are impacted by the event whilst also dealing with their own experiences of impact, thus requiring them to manage a ‘dual role’ of being both a supporter while also bereaved:

*“I had to be strong for everybody else right? I had to be strong for the students and I had to put my grieving aside so I could do that”*

[21].

Practitioners experienced the raw and immediate responses of ‘family members’ during contact with them after the person had died:

*“I remember this woman who pointed a finger at me and said I will make sure that you will never work again … this woman said you are responsible for my brother’s death. Actually your cruelty caused my brother to choose to jump through a window than be taken care of by you”*

[26].

The process of judgment was not only one-way, as participants themselves passed judgment on the ways in which family members behaved following a client’s death:

*“I just broke down and cried in the meeting [with the family] and there was nothing from them, nothing at all. This had been their daughter, and their coldness and indifference … was terribly uncomfortable, painful, and confusing”*

[29].

Experiences of ‘social and cultural norms’ were evident across six studies; four being from the US, one from Canada and one from South Korea. Participants’ fears and responses in the context of the legislative implications of a client death by suicide were evidenced for practitioners in the US and Canada:

*“I felt angry about having to consider legal implications when I was trying to deal with my own grief and help others deal with their grief”*

[29].

Legal advice might result in practitioners feeling silenced, and in turn, feeling conflicted about imposed silence:

*“There’s so much litigation that even sitting down and talking about a case you’re concerned that whatever is said might be somehow subpoenaed … some attorney will if they hear about a discussion, they’ll subpoena everyone that was there …”*

[30].

In South Korea, cultural ideas about suicide as shameful shaped the way practitioners framed and responded to the event:

*“Teachers assumed that mentioning the suicide is an insult to the deceased and their family who broke the social norms. And school community members accept it as a disgrace to the school.”*

[25].

### 3.3. Support, Learning and Living with

The category ‘Support, learning and living with’ described practitioners’ experiences both prior to and after the event and the choices and strategies that they called upon to facilitate a process of recovery, acceptance and moving forward professionally and personally. The theme of ‘Experiencing Support’ evidenced that practitioners had support needs from the moment of the suicide through the forthcoming weeks, months and even years. The inputs of others could shape the kinds of experience the practitioners had. ‘Learning’ commenced after a short time had lapsed, when reflection and review processes began to take place. ‘Living with’ was about the ways in which practitioners made choices and developed strategies that enabled them to find the best route forward from the experience. This category illustrated the diversity of experience across professions and practitioners.

#### 3.3.1. Experiences of Support

Experiences of support were evident across all studies through a wide range of experiences. Support was accessed in a variety of places; sometimes it was offered to practitioners, while others had to seek it out or accept whatever was available:

*“I had a fiancé and I had one roommate, so they gave me whatever support they could, but they weren’t professionals either … they weren’t trained in dealing with grief or anything like that”*

[21].

The ways practitioners defined ‘support’ varied across studies, with differences evident between ‘peer’, ‘professional’ and ‘formal’ support in terms of what was perceived as support and what was found to be helpful. For instance, when nurses are asked about support, they talked about peer support [20,23]. For other groups, the support received from colleagues was not seen as ‘support’ as it was not provided through a formal process:

*“No, we don’t receive any support, we’re good at supporting each other within the practice … so we have a supportive network within the practice and talk it through ourselves but we don’t have any formal back up or counselling involved.”*

[27].

There was great diversity across the studies in terms of the support that was available and offered to participants:

*“Our immediate line managers came in and we were offered basic counselling, a debriefing session immediately … we were allowed to go off work, to go home, we got follow-up phone calls at home to make sure that everything was ok and everything, and we were offered debriefing over the next few days”*

[20].

#### 3.3.2. Learning

The theme of Learning was largely evidenced in the discussion sections of the papers. It illustrated how the event of a client suicide highlighted practitioners’ gaps in and lack of knowledge, as well as their recognition of their need for training:

*“[Participants stated] that they felt “poorly prepared for a client suicide”, through their [professional] education, and that the event left them “aware of how untrained and naïve I was …”*

[28].

One study identified that as well as knowing how to support and respond to suicidal ideation and behaviour, practitioners also needed to feel personally prepared for the event of a suicide.

#### 3.3.3. Living with

This theme described the ways in which practitioners moved forward after a client suicide. As with many models of bereavement, the process was shown to be not just about ‘recovery’ but also about ‘living with’ the ongoing effects of the event:

*“I still feel sorry that she is gone. Still occasionally go over and over the events leading up to the suicide wondering if I could have done something different”*

[28].

Other practitioners found a way to mark closure of the experience either through practical rituals or cognitive processes:

*“I have acknowledged that I can not possibly save all my clients. I try to do my best always and be on top of things”*

[28].

Taking care of the self was a strategy evidenced by some practitioners, for example, giving themselves some space or time alone:

*“I take care of myself and take quiet time for myself”; “one of my personal supports is prayer and a spiritual community where we are all working on our spiritual growth”*

[22].

For some practitioners, moving on involved questioning their career options and considering the possibility of change:

*“I spent a good year seeing a career counsellor because I wasn’t sure I wanted to stay in this job”*

[21].

## 4. Discussion

The synthesised findings of 12 papers published from 11 studies describe the experiences of practitioners across a range of professional roles after a client, patient, student or service user has died by suicide. Despite the diversity of professions; the range of relationships that practitioners had with the deceased; and the global spread of the studies, the findings evidence similarity of personal and professional experience. Three categories illustrate that the experience can be horrific, shocking and has the potential to cause trauma responses; that practitioners experience intense scrutiny and perceptions of judgement and blame coming from themselves and from others; and that their experiences of support and of learning prior to and after the event shape their processes of living with the experience.

**Figure 2:**
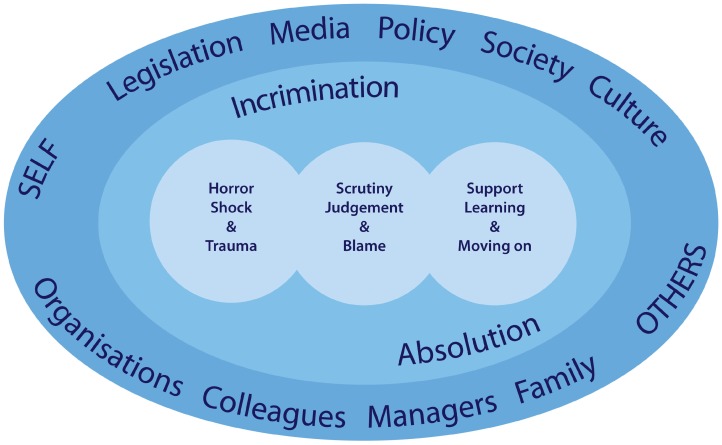
An overarching framework of the context surrounding practitioner experiences.

The framework in Figure 2 presents the overarching context within which practitioner experiences sit. It demonstrates that the complex experiences of practitioners that are described in detail in the findings of this review are not autonomous and that social processes beyond the suicide of a client shape practitioners’ experiences. There is flow and interaction between and across the three categories of findings. The experience of practitioners in any one of these categories may shape or influence their experience in the others. For instance, initial responses of shock and horror may be compounded by blaming or judgemental responses from colleagues or family members. The synthesis also highlighted that practitioners’ experiences are shaped by the social contexts within which practitioners operate. The processes of interaction between personal, professional and social contexts create the diversity and complexity of experience shown throughout these findings. The behaviours and responses of others, including colleagues, supervisors, family of the deceased as well as social and cultural factors, including legislative processes and cultural beliefs around suicide, are all important considerations within this wider context. Two further concepts were present throughout the findings of this synthesis: absolution and incrimination. Clark [42] identified that therapists experienced periods of absolution and recrimination after a client suicide. In contrast, this analysis demonstrated an experience akin to incrimination rather than recrimination; and suggests that practitioners experienced both incrimination and absolution on the part of others and themselves almost immediately following a suicide, and throughout the ongoing processing and experiencing of the events that followed in the subsequent days, weeks and months. So, it seems that the actions or behaviours of ‘others’, which may be family, managers, colleagues, organisations, are perceived as incriminating or absolving, thereby shaping practitioners’ experience within and across the three categories.

Findings from this review demonstrate a range of adverse emotional responses to a client death by suicide and therefore lend support to the idea that any individual exposed to suicide may experience a mild, moderate, or severe reaction [12,43]. Indeed, following a client death by suicide, some practitioners experienced trauma responses such as intrusive thoughts, nightmares, sleeplessness, sickness and heightened aversion to risk in their contact with clients. This aligns with previous research, which found that post-traumatic symptoms may be a response to suicide, in wider circles beyond family members, including therapists and mental health professionals [17,44]. Some of the most tangible of these trauma experiences were reported by those practitioners who were either present at the time of the death, who interacted with the body of the deceased, or who imagined what the death may have looked like. Previous research has found that train drivers who witness a death find the visual experience to be the most distressing aspect and law enforcement officers exposed to suicide scenes experience increased post-traumatic symptoms [45,46]. This suggests that visual experience may heighten trauma responses.

Practitioners may experience moral and ethical dilemmas following a client suicide and employ a number of different strategies to preserve their own wellbeing and reach a sense of absolution. A sense of moral- or value-led anguish was palpable for practitioners as they struggled to reconcile clients’ suicidal actions with their professional drive to preserve life; this was vividly evident in their self-scrutiny as they asked ‘what did I miss?’, ‘what did I get wrong?’ This self-incriminating behaviour was apparent across professions in this synthesis and appeared to deepen the sense of responsibility and blame that practitioners perceived, sometimes to a level that they felt unable to contemplate the possibility of a repeat experience, should a subsequent client die by suicide. The practitioner strived to develop strategies to preserve their own wellbeing, for instance by absolving themselves of responsibility by formulating ideas of the ‘autonomous client’ and the ‘client’s choice’ to die. These conceptualisations of client autonomy align with professional codes of ethics and practice that promote the concept of client autonomy as an important underpinning value in practice, such as the British Association for Counselling and Psychotherapy (BACP) ethical framework [47]. Narratives such as ‘you can’t save them all’ and ‘if they’re going to do it they are going to do it’ illustrate how practitioners may frame client autonomy in the instance of suicide. Testoni et al., [48] found that self-blame and other-blame were mutually excluding amongst suicide survivors who accessed support via self-help groups, that is, when survivors attention were oriented toward the social processes that may have contributed to a suicide the experience of self-blame was absent. It may be that the concept of the ‘autonomous client’ provides practitioners with the means to absolve themselves of a self-blame narrative. However, the concept of client autonomy does not appear congruent with a sense of blaming the client; rather practitioners, in this review, seemed to be seeking a means of accepting or even feeling compassion for their clients’ actions. However, the ‘autonomous client’ concept presents a problem if we are to understand that people who behave in suicidal ways are not operating from a place of full autonomy; and, therefore, suicide is not a choice [49]. This leaves a gap in the narrative for a new ‘story’ to be developed that both mitigates the practitioner from misplaced blame and acknowledges that the suicidal individual may be highly distressed and as such, compromised in making clear and rational autonomous decisions. To move away from ideas of blame, Clarke [49] suggests a narrative of ‘understandability’ and ‘respect for the person’, which feels more comfortably aligned with the processes demonstrated by practitioners in this synthesis. Practitioners also seek to absolve themselves of the potential for future client death by suicide. This might include the adoption of practice habits that identify subsequent clients as risky and engaging in choices and behaviours that affect clients’ experience of the service, even affecting their treatment through early hospitalisation [29]. This aligns with previous findings that suggest the impact of client suicide on professional practice may affect clinical assessment and treatment decisions including changes in assessing suicide risk, the frequency of referrals to other colleagues and choices of treatment including increased hospitalisation [50].

The findings of this review demonstrate that postvention responses are needed for this population of practitioners. In contrast, a significant narrative in the postvention literature situates practitioners as the providers of postvention and support rather than as those who are impacted and potentially in need of support themselves [51,52]. Those who have been impacted by a suicide death are often identified in the role of ‘client’, that is, the person who will be supported by a practitioner. The practitioner who has been impacted and who needs to be supported appears to be largely absent in the research literature about postvention need and provision. National postvention guidelines in the UK and US are underpinned by the Continuum of Suicide Survivorship model, with recommended responses guided according to the level of impact experienced by the individual [12]. In the UK, guidelines suggest that practitioners are likely to sit in the ‘affected’ category of the continuum, and recommend that all people bereaved or affected by suicide should at the very least be given guidance via a ‘Help is at Hand’ information leaflet or signposting to sources of support [53,54]. US national guidelines identify that everyone exposed to a suicide should be offered some level of postvention support; and this should be offered around three levels of care relating to immediate needs; ongoing support and clinical treatment [55]. It is clear that whilst these guidelines recognise the potential for trauma responses at all levels of exposure to suicide, they are constructed around the experience of the individual. This review has shown how social and cultural contexts may shape the individuals experience and, as such, it is important that postvention responses are rooted in these same contexts so that they are fit for purpose to meet need within diverse social and cultural settings. For instance, in settings where there are greater taboos and stigma associated with suicide, it may be important not to be led by prescriptive generic, usually western, ideas of ‘what works’ [56]. It might be suggested therefore, that responses and support strategies could be developed locally. Indeed, Ting et al., [30] appeared to evidence how team cultures and the kinds of postvention support offered to practitioners may help them in developing a stronger sense of clarity that client actions and choices are distinct from practitioner actions and choices, which may be helpful in practitioners’ own process of reconciling the event. Given that the findings of this synthesis situate the practitioners’ experience within social contexts, this raises an opportunity for teams, managers and organisations to play a role in developing targeted and nuanced postvention responses that are specific to practitioner roles, practitioner relationships with clients and to practice settings. These findings align with Grad’s [57] guidelines for mandatory and optional postvention responses for assisting clinical staff after a suicide.

As well as providing postvention support for practitioners, organisations may also have a role in preparing practitioners for the event of a client suicide. We know that practitioners have a higher likelihood than the general population of encountering suicide death that raises the question of whether support could begin before an event occurs [6,7,8,9,10,11]. This may take the form of preparing/training practitioners for a potential client suicide; talking about the possibility that it may happen and what it may be like, and what the organisational postvention policy and procedures would be. Juhnke and Granello [58] presented a six-point ‘pre-suicide preparation plan’ for Mental Health Practitioners; the plan addressed the things that can be put in place before an event to support practitioners if a suicide should happen, so, in essence, it is a postvention plan. However, the existence of a set of strategies may also nurture a culture in which exploratory conversations can take place before a suicide death occurs. Given the findings of this review, it might be suggested that pre-suicide preparation takes the form of educating practitioners ahead of the potential event around expected impact, strategies for coping and helpful narratives with the aim of supporting them to avoid the pitfalls of self-blame and incrimination.

### 4.1. Strengths and Limitations

This is the first review to bring together and synthesise the qualitative literature that reports the experiences of health, social care and education practitioners following the death by suicide of a client, patient, student or service user. This review adds to current knowledge of the experiences and postvention needs of this group of people after a suicide. The synthesis developed findings that moved beyond the individual experiences reported in the studies to situate those experiences socially and culturally. A strength of this review lies in the rigorous methodology applied to searches, quality appraisal, analysis and synthesis, guided by the ENTREQ statement [35]. However, findings must be considered in the context of the limitations of the study. This review set out to synthesise qualitative literature with a focus on experiences and perceptions, and as such has not included studies that considered causal relationships. Whilst the studies included in the review are situated globally, it is worth noting that four of the studies were undertaken in the US; and that two of the papers came from a single study in Canada. The review also only included studies published in the English language and is limited by the exclusion of research published in other languages. The diversity of professions, organisational settings and cultural contexts may also be seen as a limitation in terms of enabling generalisations to be made in the analysis; however, they identified a number of unifying experiences and together generated a synthesis that highlighted how social and cultural contexts shape individual experience.

### 4.2. Implications and Recommendations

Pre-suicide training should be provided to practitioners to prepare them for the kinds of thoughts, feelings and behaviours that they might experience following a client suicide. Postvention response and support policies should be developed that recognise that practitioners may also be potentially impacted by a client suicide: where the aim should be to mitigate trauma symptoms, adverse emotional responses and any subsequent adverse impact on future practice; and support be provided that is sensitive to the social and cultural context within which a suicide occurs and the ethical and organisational cultures in which practitioners are operating.

The challenges in contextualising the findings of this study point toward a need for broader and deeper understanding of the nuanced experiences of practitioners following a suicide. Qualitative studies would be well placed to explore the experiences of practitioners that have not previously been researched; for instance, by focusing on the experiences of specific professionals such as education providers, or within specific settings such as Higher Education. Future research might also explore the experiences of trauma as opposed to bereavement following suicide with a focus on the wider networks that are exposed to or affected by a suicide. The processes of incrimination and absolution were identified in this review as impacting practitioners experience; the authors are not aware of these processes being previously identified in postvention literature, and further exploration of them and of their potential to shape experience would add to knowledge in this area. A meta-analysis of the quantitative literature that addresses the impact of suicide on practitioners would increase knowledge and contextualise the findings of this review by providing a more holistic overview of practitioner experiences.

## 5. Conclusions

This review explored the existing qualitative literature that reported on health, social care and education practitioners’ experiences following the death by suicide of a client, patient, service user or student. This review highlighted both commonality and diversity of experience and has contextualised those experiences socially and culturally, by acknowledging the potential influence of settings in which practitioners operate and experience client suicide. The responses and behaviours of diverse ‘others’ are perceived by practitioners as incriminating or absolving and this can shape the ways in which a suicide is experienced. Within the broader postvention literature, the practitioner is often identified as a postvention provider supporting others who are impacted by suicide; this review demonstrated that practitioners can experience traumatic and adverse emotional responses to a suicide and that targeted postvention is needed to support practitioners in processing the impact and in developing narratives that enable continued safe practice. Postvention practice and policies should be contextualised socially, culturally and organisationally so that they are sensitive to individual need.

## Figures and Tables

**Table 1:** Attributes of included studies.

Author and Date	Location of Research	Participant Population and Number	Aim of Study	Data Collection	Data Analysis	Results/Findings
Bohan and Doyle (2008) [20]	Ireland	Psychiatric nurses on acute inpatient units within three large hospitals *N* = 9	To describe psychiatric nurses’ experience of suicide and suicide attempts in an acute unit and explore their perceptions of the support they received after the incident.	Semi-structured interviews—audio taped and transcribed	Burnard’s [39] method of data analysis – detailed systematic description of themes.	Four themes: Nurses’ experiences of patient suicide/suicide attempts Nursing care following an incident of suicide/suicide attempt Feelings experienced by nurses following a suicide/suicide attempt Support for nurses following a suicide/suicide attempt
Christianson and Everall (2008) [21]	Canada	School Counsellors *N* = 7	To gain an in-depth understanding of school counsellors experiences of client suicide from their perspective.	Telephone semi-structured interviews. Digitally recorded and transcribed.	Grounded Theory	Three themes related to training, resources and self-care: National training/practice standards Support resources Self care
Christianson and Everall (2009) [22] Reports the same study as Christian and Everall (2008) [21]	Canada	School Counsellors *N* = 7	To explore the experiences of school counsellors who had lost clients to suicide. Qu’s = ‘What are school counsellors’ experiences of client suicide?’ ‘What impact do participants believe client suicide had on their lives?’	Telephone interviews (geographically diverse population) – two interviews per participant.	Grounded Theory	Four themes: Taming the control beast Wearing the mask Interpreting the dance Staying in the game
Darden and Rutter (2011) [23]	US	Clinical Psychologists *N* = 6	An in-depth exploration of the clinician’s experience in losing a client to suicide.	In-person semi-structured interview.	Consensual qualitative research (CQR) methods followed—themes, domains and categories.	Six domains: Psychologist’s view of suicide Clinical aspects of the case The suicide Impact Recovery Client’s Family
Davidsen (2010) [24]	Denmark	General Practitioners *N* = 1 4	To investigate how GPs were affected by patients’ suicides and whether their reaction was linked to their inclination to explore suicide risk in the patient who died by suicide, and whether the GP’s current inclination to explore suicide risk has been influenced by their experience of a patient death by suicide.	Semi structured interviews—conducted as part of larger study (Davidsen, 2009) [40]	Interpretative Phenomenological Analysis (IPA)	Super-ordinate theme: patients’ suicides. Underlying themes: Emotional impact Self-scrutiny Talking about suicide
Kim (2019) [25]	Korea	School Teachers *N* = 5	To explore the bereavement experiences of teachers and the challenges they face in coping with student suicide.	Semi-structured interviews	Colaizzi’s [41] Phenomenological approach	Four themes: Examination of the suicide Suspension of grief Tolerance of the suicide Renewed perception of role in preventing student suicide
Matandela and Matlakala (2016) [26]	South Africa	Nurses in General Hospital *N* = 6	To present the experience of nurses who cared for patients who died by suicide while admitted in a general hospital	Interviews audio recorded and transcribed	Manual general qualitative content analysis.	Five themes: Experience of disbelief and helplessness Feelings of blame and condemnation Feelings of guilt and inadequacy Emotional reaction Fear of reprisal
Saini et al., (2016) [27]	England	General Practitioners *N* = 198	To explore GPs views on how they are affected by a patient suicide and the formal support available to them following a patient suicide.	Semi-structured interviews, audio recorded and transcribed.	Descriptive statistics and a framework thematic approach	Three inter-related themes: Part and parcel Failing patients Informal support systems
Sanders et al., (2005) [28]	US	Mental Health Social Workers *N* = 145 Sample taken from a larger quantitative study—this sample being all participants who responded that they had experienced a completed client suicide.	To expand the understanding of the reactions of social workers to client suicide. Three research questions: 1. What professional and personal reactions do social workers experience immediately following a client suicide completion? 2. What professional and personal reactions do social workers experience long term, following a client suicide completion? 3. What is the relationship between time since the client suicide completion and the social workers’ reactions? The first two being relevant for this study.	Two open-ended questions at the end of a questionnaire. • Please describe how you felt in the seven days immediately following the client suicide. • Please describe how you feel now when you think about the client suicide.	Coding and constant comparative methods by two researchers working independently and comparing their results. Reviewed by third researcher.	Major themes immediately following client suicide: Deep sadness and depression Trauma and shock Feelings of professional failure Anger and Irritability Self blame Worries and Fear Major themes at time of survey: Continued emotional reactions Changes in practice Reconciliation Power and control issues Nothingness
Tillman (2006) [29]	US	Psychoanalysts/psychoanalytic psychotherapists *N* = 12	Interview question: ‘I am conducting a study about the effect of patient suicide on clinicians; I am interested in how this event has affected you. Would you tell me, in as much detail as possible about you’re your experience?’	Semi-structured interviews. Transcribed and audio recorded.	Coded by two researchers—using a psychoanalytic lens—‘a synthesis was made of the categories to arrive at a ‘best fit’ thematic analysis.’	A research vignette is presented in the paper to ‘illustrate the depth and range of experiences reported by the clinicians’ Eight themes: Traumatic responses Affective responses Treatment specific relationship Relationships with colleagues Risk management Grandiosity, shame, humiliation, guilt, judgement, blame A sense of crisis Effect on work with other patients Sit within three domains Traumatic loss and grief Interpersonal relationships Professional identity concerns
Ting et al., (2006) [30]	US	Mental Health Social Workers *N* = 25	What are the reactions experienced by a group of mental health social workers after a client suicide.	Semi-structured telephone interviews. Audio recorded and transcribed.	Constant comparative method with open coding.	Twelve Themes: Denial and Disbelief Grief and Loss Anger at client Agency and society Self-blame and guilt Professional failure and Incompetence Responsibility Isolation Avoidant behaviours Intrusion Change in professional behaviour changes in practice Changes in the professional environment Justification Acceptance
Wang et al., (2016) [31]	China	Nurses in a General Hospital *N* = 15	To explore the impact of inpatient suicides on nurses working in front-line, the patterns of regulation and their needs for support.	Semi-structured in-depth interviews	Colaizzi’s seven-step phenomenological method by two interviewers.	Four ‘centre themes’ and associated ‘sub-themes’ were identified. Nurses’ cognition about inpatient suicide Inpatients are at a high risk of suicide Inpatient suicide is difficult to prevent Shortage of suicide preventing skills Psychological reaction Shock and panic Sense of fear Self-accusation or guilt Frustrated or self-doubt Impact on practice Stress Excessive vigilance Burnout Patterns of regulation Pouring out bitterness Avoidance

**Table 2:** Categories, themes and concepts.

Category	Theme	Concept
Horror, Shock and Trauma	Witnessing suicide	The horror
		In the moment responses
		Shock and trauma
	Responses to suicide	Loss and grief
		Mind and body responses
	Dealing with suicide	Connections and closeness
		Absence and distance
		The dual role
Scrutiny, Judgment and Blame	Thinking about responsibility	Am I responsible?
		The un/expected death
		Professional failure—guilt, reprisal and reputation
		The autonomous client
	Having to carry on	Aloneness
		Issues of control
		Avoidance strategies
		Hyper-vigilance
	Dealing with others	The organization and colleagues
		The family
		Cultural and social norms
Support, Learning and Living with	Experiences of support	
	Learning	
	Living with	

## Supplementary Materials

The following are available online at https://www.mdpi.com/1660-4601/16/18/3293/s1, Table S1: CASP appraisal of articles.
