# Supplementary material for: What Is the Experience of Practitioners in Health, Education or Social Care Roles Following a Death by Suicide? A Qualitative Research Synthesis
Source: Int J Environ Res Public Health. 2019 Sep 7;16(18):3293. doi: 10.3390/ijerph16183293 (PMC6766076; doi:10.3390/ijerph16183293)
Supplement: Supplementary file 1 [file ijerph-16-03293-s001.zip › ijerph-587234-supplementary.docx]

**Supplementary Table S1:** CASP appraisal of articles.

|  | **Bohan & Doyle (2008)** | **Christianson & Everall (2008)** | **Christianson & Everall (2009)** | **Davidsen (2011)** | **Darden & Rutter (2011)** | **Kim (2019)** | **Matandela & Matlakala (2016)** | **Saini et al. (2016)** | **Sanders et al. (2005)** | **Tillman (2006)** | **Ting et al. (2006)** | **Wang et al. (2016)** |
| --- | --- | --- | --- | --- | --- | --- | --- | --- | --- | --- | --- | --- |
| 1. Statement of Aims | Yes | Yes | Yes | Yes | Yes | Yes | Yes | Yes | Yes | Yes | Yes | Yes |
| 2. Appropriate methodology | Yes | Yes | Yes | Yes | Yes | Yes | Yes | Yes | Yes | Yes | Yes | Yes |
| 3. Appropriate design | Yes | Yes | Yes | Yes | Yes | Yes | Yes | Yes | Yes | Yes | Yes | Yes |
| 4. Appropriate recruitment strategy | Yes | Yes | Yes | Yes | Yes | Yes -Acknowledgment of challenges of recruiting teachers into the study | Yes | Yes | Ppts selected from a larger quant study – being the 145 ppts who had experience of a completed client suicide. | Not described | Yes – Ppts responded to an earlier quant study and indicated their interest in this study. Previous study anonymous so no ability to check whether a representative sample from the whole. | Yes |
| 5. Data collection method justified and clear | Yes | Yes | Yes | Yes | Yes | Yes | Yes | Yes | Yes | Yes | Yes | Yes |
| 6. Consideration of relationship b/tw researcher & ppts | N | N | N | N | States details of team members and acknowledges that team members have no experience of client suicide | Acknowledgement that the researcher is a teacher | N | N | N | N | N | N |
| 7. Consideration of ethical issues | Yes – Approval sought from academic institution and sites of data collection | Yes | Yes | No | No | Yes | Yes | Yes | No | No | No | Yes |
| 8. Rigorous data analysis | There is no mention of oversight by other researchers | Yes | Yes | Yes | Yes | Yes | Yes | Yes | Yes | Yes | Yes | Yes |
| 9. Clear statement of findings | Yes | Yes – reported findings relating to training & practice standards, support resources and self-care. Additional findings relating to more personal impact are reported in Christianson & Everall (2009). | Yes | Yes | Yes | Yes | Yes | Yes | Yes | Yes | Yes | Yes |
| 10. Is the research valuable? | Yes | Yes | Yes | Yes | Yes | Yes | Yes | Yes | Yes | Yes | Yes | Yes |
| Date of Appraisal | 15.03.18 | 22.03.18 | 21.03.18 | 13.03.18 | 16.03.18 | 11.04.19 | 12.03.18 | 14.03.18 | 21.03.18 | 21.03.18 | 20.03.18 | 15.03.18 |
